# Novel Carbon Dots Derived from *Glycyrrhizae Radix et Rhizoma* and Their Anti-Gastric Ulcer Effect

**DOI:** 10.3390/molecules26061512

**Published:** 2021-03-10

**Authors:** Yuhan Liu, Meiling Zhang, Jinjun Cheng, Yue Zhang, Hui Kong, Yan Zhao, Huihua Qu

**Affiliations:** 1 School of Traditional Chinese Medicine, Beijing University of Chinese Medicine, Beijing 100029, China; lyhown@163.com (Y.L.); 18811790361@163.com (M.Z.); carlosjjcheng@163.com (J.C.); doris7629@163.com (H.K.); zhaoyandr@gmail.com (Y.Z.); 2 School of Life Sciences, Beijing University of Chinese Medicine, Beijing 100029, China; 201801024@bucm.edu.cn; 3 Center of Scientific Experiment, Beijing University of Chinese Medicine, Beijing 100029, China

**Keywords:** *Glycyrrhizae Radix et Rhizoma*, carbon dots, acute alcoholic gastric ulcer, protective effect

## Abstract

*Glycyrrhizae Radix et Rhizoma* (GRR) is one of the commonly used traditional Chinese medicines in clinical practice, which has been applied to treat digestive system diseases for hundreds of years. GRR is preferred for anti-gastric ulcer, however, the main active compounds are still unknown. In this study, GRR was used as precursor to synthesize carbon dots (CDs) by a environment-friendly one-step pyrolysis process. GRR-CDs were characterized by using transmission electron microscopy, high-resolution TEM, fourier transform infrared, ultraviolet-visible and fluorescence spectroscopy, X-ray photoelectron spectroscopy, X-ray diffraction and high-performance liquid chromatography. In addition, cellular toxicity of GRR-CDs was studied by using CCK-8 in RAW264.7 cells, and the anti-gastric ulcer activity was evaluated and confirmed using mice model of acute alcoholic gastric ulcer. The experiment confirmed that GRR-CDs were the spherical structure with a large number of active groups on the surface and their particle size ranged from 2 to 10 nm. GRR-CDs had no toxicity to RAW264.7 cells at concentration of 19.5 to 5000 μg/mL and could reduce the oxidative damage of gastric mucosa and tissues caused by alcohol, as demonstrated by restoring expression of malondialdehyde, superoxide dismutase and nitric oxide in serum and tissue of mice. The results indicated the explicit anti-ulcer activity of GRR-CDs, which provided a new insights for the research on effective material basis of GRR.

## 1. Introduction

*Glycyrrhizae Radix et Rhizoma* (GRR), named Gan Cao in Chinese, is one of the most commonly used herbs in clinical practice, first recorded in the *Prescriptions for Fifty-two Diseases*. GRR has been widely used to treat digestive diseases such as weakness of spleen and stomach, bad appetite, abdominal pain and peptic ulcer for more than 2000 years in traditional Chinese medicine. Some scholars summarized Professor Liu Fengbin’s clinical experience and academic thoughts of chronic atrophic gastritis and found that GRR was one of the 10 core herbs for gastroesophageal reflux disease treatment [1]. The modern pharmacological studies have also demonstrated that GRR have the activity of anti-ulcer, anti-inflammatory, anticancer, antibacterial and antioxidant [2,3,4]. In addition, GRR belongs to the medicine homologous food, which is also very common in daily diet [5].

Gastric ulcer is a common chronic disease with long course and easy recurrence, which has a high incidence among heavy drinkers [6]. Through the collection and collation of literature, it was found that GRR was one of the most frequently used traditional Chinese medicine in the treatment of gastric ulcer, and experimental research had also confirmed that some components in GRR had a good anti-ulcer effect. There were some researchers had studied the main active compounds of anti-ulcer in GRR. Bennett’s [7] experimentz on rats with gastric mucosal injury showed that GRR had an anti-ulcer activity, and Ishii’s [8] further study found that the mechanism might be related to its ability to reduce gastrin release. However, the anti-ulcer material basis of GRR is still controversial.

A comprehensive review of medical documents in different periods showed that the processing methods of GRR had undergone a process from simple to complex. With the rapid development of medicine, the processing technology and auxiliary materials of GRR had been recorded in detail, which could be flexibly applied to the treatment of various diseases. Although there were many processing methods of GRR, the changes of material basis after high temperature calcination were not clear. In recent years, researchers had tried to explain the mechanism of GRR’s processing from the perspective of small molecular compounds, but the results were still inconclusive. Therefore, restoring the original processing method of GRR and further studying of material basis and pharmacological effect are indispensable links to explore its processing mechanism.

It is noteworthy that the high temperature calcination of GRR is similar to the preparation process of the carbon dots (CDs), a kind of dispersive spherical fluorescent carbon nanoparticle smaller than 10 nm [9,10,11]. Due to the characteristics of adjustable luminescent range, large two-photon absorption cross-section, good light stability, easy functionalization, low toxicity and good biocompatibility, CDs have a good application prospect in the fields of biomarkers and drug carriers [12,13,14,15,16]. Currently, the intrinsic bioactivities of CDs is gradually gaining attention. Our group had found and successfully prepared CDs from a variety of charcoal drugs [17,18]. Therefore, we wonder whether this nano-component also exists in calcined GRR and relates to its anti-ulcer effect.

In this paper, we report the preparation process of a new type of CDs through calcination, decocting and dialysis from the GRR, which was named GRR-CDs. Furthermore, we characterized the GRR-CDs by using transmission electron microscopy (TEM), high-resolution TEM (HRTEM), X-ray photoelectron spectroscopy (XPS), X-ray diffraction (XRD), fluorescence analysis (FL), ultraviolet-visible (UV-Vis), fourier transform infrared (FT-IR) spectroscopy and high-performance liquid chromatography (HPLC). Then we estimated the cytotoxicity and anti-ulcer activity of the GRR-CDs.

## 2. Results

### 2.1. Characterization of GRR-CDs

**Figure 1:**
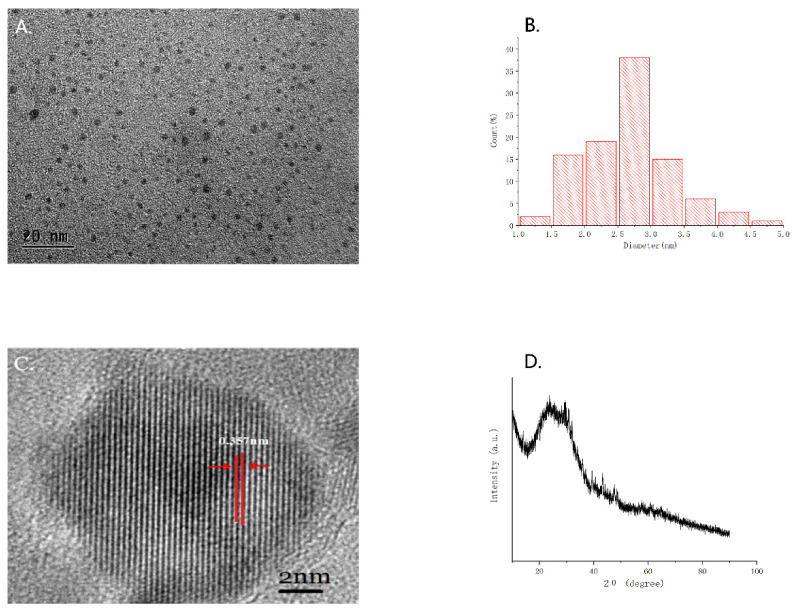
(**A**) Transmission electron microscopy (TEM) images of GRR-CDs displaying ultra-small particles. (**B**) Histogram depicting particle size distribution. (**C**) High-resolution TEM (HRTEM) image of GRR-CDs. (**D**) XRD pattern.

As shown in the TEM image (Figure 1A), The GRR-CDs were the uniformly distributed spherical structure, and their particle size distributed between 1–5 nm (Figure 1B). Furthermore, the HRTEM (Figure 1C) showed that the GRR-CDs had a lattice spacing of 0.357 nm, and the XRD (Figure 1D) pattern showed a distinct diffraction peaks (2θ = 23.02°), which was attributed to amorphous carbon composed in a considerably random fashion. The structural characteristics of GRR-CDs were similar to those of nano components CDs.

**Figure 2:**
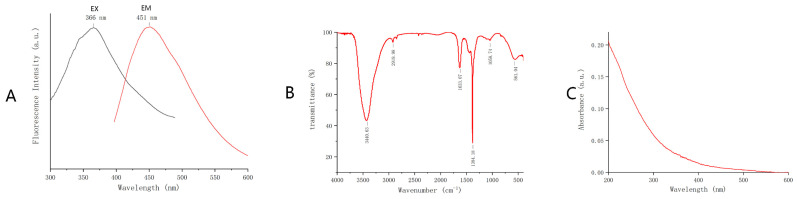
(**A**) Fluorescence spectrum. (**B**) FTIR spectrum. (**C**) Ultraviolet-visible (UV-vis) spectrum.

The FL spectrum showed that the maximum excitation wavelength of GRR-CDs was 366 nm and the maximum emission wavelength was 451 nm (Figure 2A). The FQY was measured to be 2.51% with quinine sulfate as the standard solution and 366 nm as the excitation wavelength.

The purified GRR-CDs FTIR spectrum (Figure 2B) showed the characteristic peaks at 3441, 2920, 1633, 1384 and 1059 cm^−1^. The broad absorption peak around 3441 cm^−1^ was O-H characteristic peak, the C-H groups were indicated by the sharp peak at 2920 and 1384 cm^−1^, and the weak C-O stretching band occurred at 1059 cm^−1^.

In addition, The UV-Vis absorption spectrum (Figure 2C) showed a broad absorption peak between 200 nm and 600 nm, without any evident peak.

**Figure 3:**
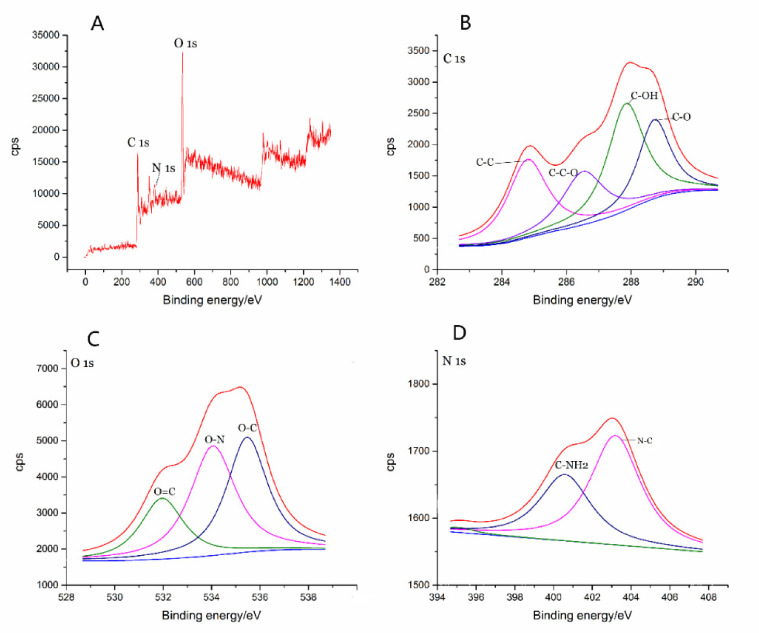
(**A**) X-ray photoelectron spectroscopy (XPS) survey spectra. (**B**) C 1s, (**C**) O 1s and (**D**) N 1s XPS spectra.

As shown in the X-ray photoelectron spectroscopy (Figure 3A), the GRR-CDs contained C, O, N, P and S elements, among which C and O elements had the highest contents, accounting for 53.95% and 40.28% of the total atomic weight, respectively. C 1s could be divided into four peaks (Figure 3B), corresponding to C-C, C-C-O, C-OH and C=O groups; O 1s could be divided into three peaks (Figure 3C), corresponding to O=C, O-N, and O-C groups; N 1s could be divided into two peaks (Figure 3D), corresponding to C-NH2 and N-C groups.

### 2.2. Fingerprint Profile of GRR and GRR-CDs

**Figure 4:**
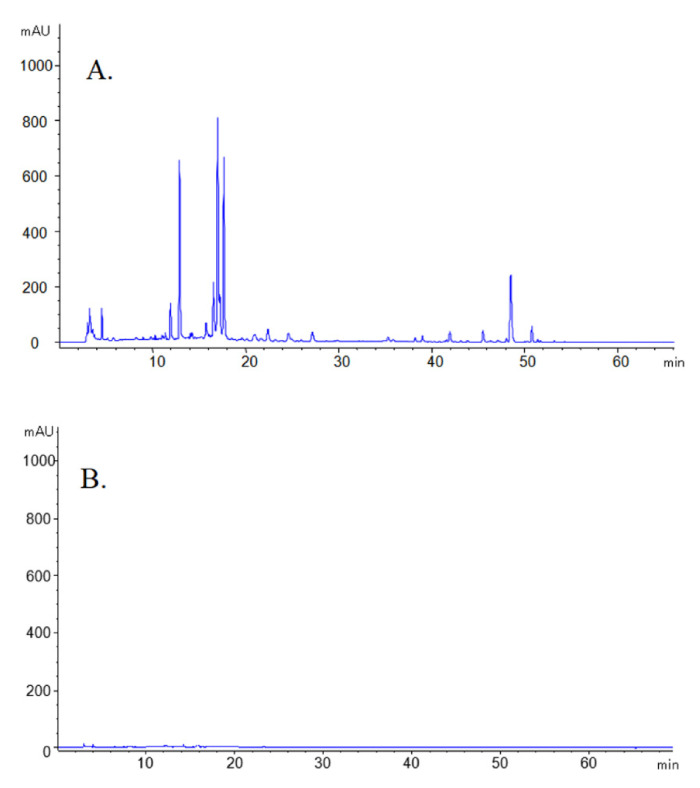
High performance liquid chromatogram of GRR (**A**) and GRR-CDs (**B**) aqueous solution.

The HPLC results of GRR and GRR-CDs aqueous solution were shown in Figure 4. The GRR aqueous solution had complex components and contains a variety of small molecules such as liquiritin apioside, glycyrrhizic acid and so on (Figure 4A). Under the same conditions, the HPLC of GRR-CDs showed no characteristic peaks of these substances (Figure 4B).

### 2.3. Cellular Toxicity of GRR-CDs

**Figure 5:**
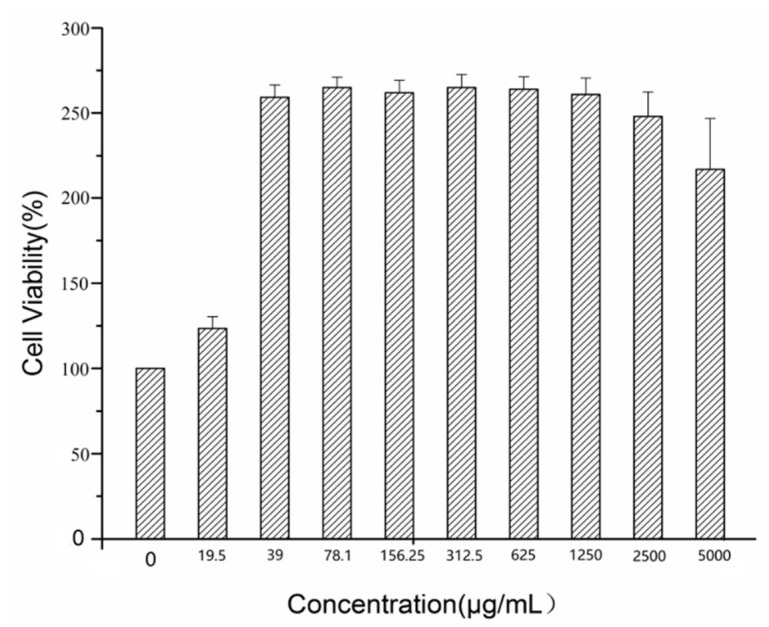
Effect of different concentrations of GRR-CDs on cell viability.

Figure 5 showed the viability of RAW 264.7 cells treated with GRR-CDs in the concentration range from 19.5 to 5000 μg/mL for 24 h. Within this range, GRR-CDs did not inhibit cell growth, and when the concentration exceeded 1250 μg/mL, the effect of increasing cell activity was weakened.

### 2.4. Anti-Gastric Ulcer Activity of GRR-CDs

The gastric ulcer index and inhibition rate of each group were shown in the Table 1. The gastric ulcer index of the model group was significantly higher than that of the control group (*p* < 0.01), which indicated that the acute alcoholic gastric ulcer model was successfully established. Compared with model group, high-, medium- and low-dose GRR-CDs could significantly reduce the gastric ulcer index and improve the inhibition rate, but there was no statistical difference among the three groups.

**Figure 6:**
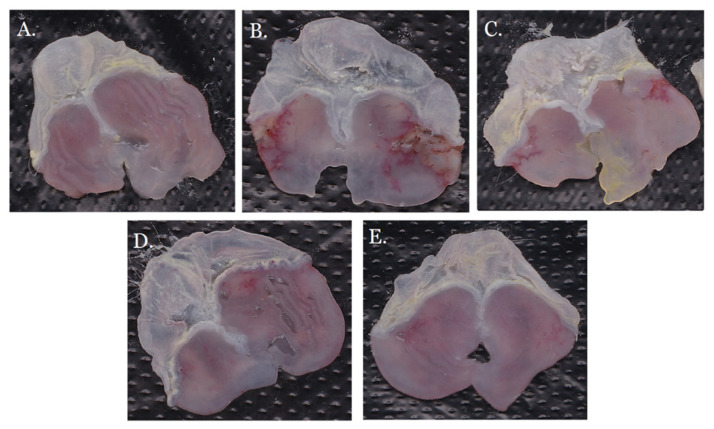
Gastric mucosal injury of mouse in (**A**) control group, (**B**) model group, (**C**) GRR-CDs high-dose group, (**D**) GRR-CDs medium-dose group and (**E**) GRR-CDs low-dose group.

As shown in Figure 6A, the color and morphology of the gastric tissues in the control group were normal, and the gastric mucosas were homogeneous and intact. In the model group (Figure 6B), there were a lot of linear ulcers and obvious white moss in the gastric mucosas. The ulcer degree of GRR-CDs low-dose group (Figure 6E) was significantly lower than that of the model group, with only a small range of linear and dot ulcers. The GRR-CDs high-dose group (Figure 6C) had small areas of linear ulcers. The GRR-CDs medium-dose group (Figure 6D) had the best antiulcer effect, with no obvious linear ulcers and only a few ulcer spots.

**Figure 7:**
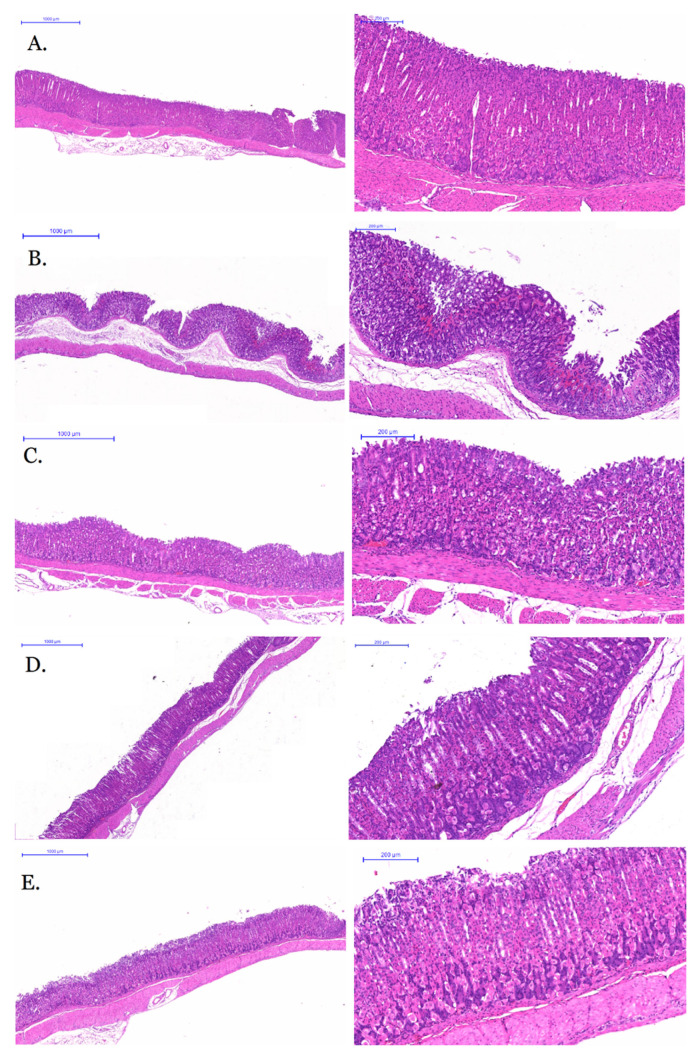
Morphological section of mouse gastric tissue of (**A**) control group, (**B**) model group, (**C**) GRR-CDs high-dose group, (**D**) GRR-CDs medium-dose group and (**E**) GRR-CDs low-dose group.

The morphological section of mice gastric tissue were shown in Figure 7. The gastric tissue cells in the control group (Figure 7A) were normal and arranged regularly, without obvious inflammatory cell infiltration and red blood cell overflow, and gastric mucosas were smooth without obvious defect. The gastric mucosas of model group (Figure 7B), under the low power microscope, were damaged in a large area with a deep degree. At the high-power microscope, a large number of red blood cells overflowed from tissues, inflammatory cells infiltrated digestive glands, and abnormal cell morphology could be seen in the tissues. The gastric mucosas in the GRR-CDs high-dose group (Figure 7C) were not smooth, but the lesions were minor, and no ulcer scar was formed. The cell morphology was basically normal and regular, with no obvious red blood cell overflow. Gastric mucosas in GRR-CDs medium-dose group (Figure 7D) were relatively smooth with a little of injury and inflammatory cell infiltration. The lesion area of the GRR-CDs low-dose group (Figure 7E) was larger than that of the high and medium-dose groups, but only the gastric mucosas were damaged, and the secretory gland and other tissues and cells were not injured.

### 2.5. Effect of GRR-CDs on Biochemical Indexes in Serum and Tissues

**Figure 8:**
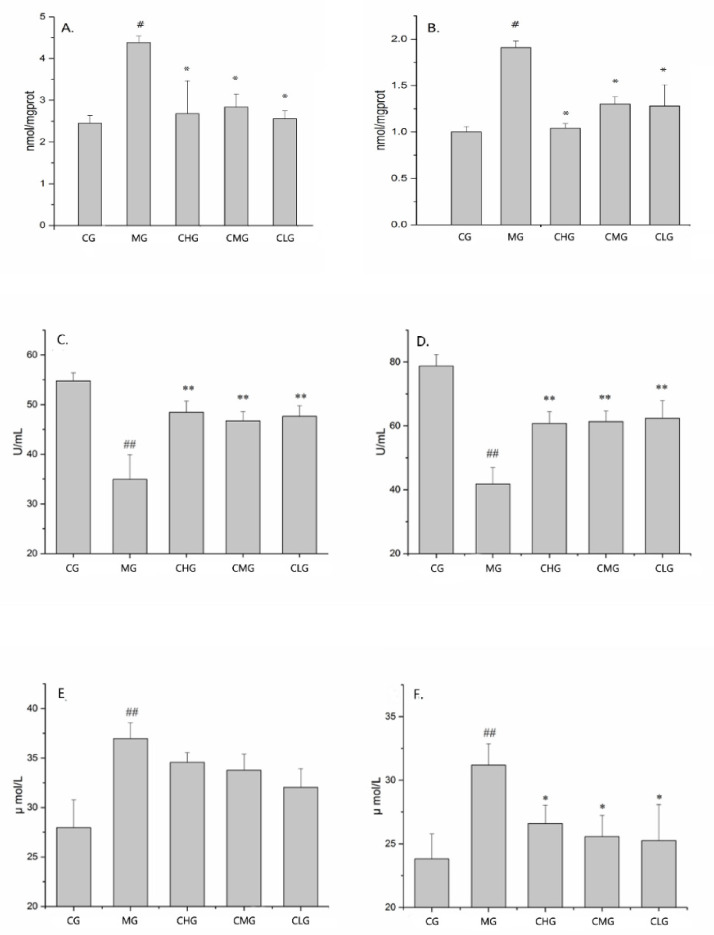
Effect of GRR-CDs on biochemical indexes. (**A**) MDA content in serum, (**B**) MDA content in gastric tissue, (**C**) SOD content in serum, (**D**) SOD content in gastric tissue, (**E**) NO content in serum and (**F**) NO content in gastric tissue. Groups (*n* = 9 per group): control group (CG), model group (MG), GRR-CDs high-dose group (CHG), GRR-CDs medium-dose group (CMG) and GRR-CDs low-dose group (CLG). ^#^ *p* < 0.05 and ^##^ *p* < 0.01 compared with control group, * *p* < 0.05 and ** *p* < 0.01 compared with model group.

As shown in the Figure 8A–D, the MDA and SOD content in serum and gastric tissues of the control group were statistically different from that of the model group (*p* < 0.05). Compared with the model group, the GRR-CDs high-, medium- and low-dose groups all had a positive effect (*p* < 0.05) but there were no significant difference among the three groups (*p* > 0.05), which was consistent with the experimental results of anti-gastric ulcer activity.

Figure 8E,F showed the content of NO in serum and gastric tissue of each group. Compared with the control group, the NO content in gastric tissue and serum of the model group decreased significantly (*p* < 0.05). The GRR-CDs high-, medium- and low-dose groups all had a positive effect (*p* < 0.05) on the gastric tissue, but there was no effect on the serum in three medicated groups (*p* > 0.05).

## 3. Discussion

Since ancient times, GRR has been widely used in the treatment of various diseases, especially in spleen and stomach. The processing of GRR has always been a hot spot in clinical application and modern research. It had been reported that the traditional processing method of GRR was to carbonize it into varying degrees. According to the literature, the CDs could be processed from some herbs and plants [19], and the preparation method of high temperature calcination was similar to that of GRR, which led us to wonder if carbonized GRR contains similar CDs. In preliminary work, we had demonstrated that CDs was the material basis of hemostatic bioactivity of charcoal chinese drugs, such as *Pollen Typhae* carbonisata [20], *Phellodendri Cortex* carbonisatus [21], *Junci Medulla* carbonisata [22], etc. Moreover, our group had also found some other curative effect of carbon nano-ingredients, for example, *Lonicerae Japonicae Flos* carbonisata-derived CDs were found to have thermoregulatory effects [23], *Aurantii Fructus Immaturus* carbonisata-derived CDs possess antihyperuricemic and anti-gouty arthritis activities [17]. Therefore, our further research will focus on the other efficacy of charcoal-processed drugs.

In this study, we found a nano-component in the water decoction of carbonized GRR by calcining, decocting and dialyzing the crude GRR. This active component that did not exist in the crude GRR was named as the GRR-CDs. Based on the preparation and characterization methods of nanomaterials, the morphology features, optical properties, elemental composition and surface-active groups of GRR-CDs were analyzed by using TEM, HRTEM, XRD, XPS, UV–Vis, FL and FTIR, respectively. Concurrently, HPLC was used to compare and evaluate the aqueous solutions of GRR and GRR-CDs, and it was found that GRR-CDs did not contain small molecules compounds which was generally considered to be the material basis for the efficacy of Chinese medicine. This result was consistent with our previous research that the active ingredient of charcoal drugs was the remaining carbonisata-derived CDs after removing of the small molecule compounds [24,25].

Cell proliferation and toxicity experiments showed that GRR-CDs had no cytotoxicity in the concentration range of 19.5–5000 nm/mL. The result indicated that GRR-CDs was an economical, environmentally friendly and excellent safe resource of traditional Chinese medicine.

The pathogenesis of gastric ulcer is complex and unclear. Previous studies had shown that gastric ulcer was mainly caused by the imbalance between aggressive factors and defensive factors, and alcohol is one of the typical factors that can lead to gastric mucosal injury [26]. Alcohol is first digested by the gastrointestinal tract and then absorbed into the blood. Therefore, high concentration of alcohol intake will first erode the gastric mucosa, destroy the protective layer through the digestion of gastric mucus and bicarbonate, which will result in irreversible damage and then induce acute gastric ulcer. Pathological study showed that the pathogenesis of acute alcoholic gastric ulcer was closely related to neutrophil infiltration, proinflammatory factor release and oxidative stress [27,28]. Therefore, the acute gastric ulcer model of mice was established by intragastric administration of high concentration alcohol. The histological characteristics, ulcer morphology, healing and recurrence process of this model is similar to that of human gastric mucosa injury, and the model is easy to establish with a good repeatability. Through the investigation of gastric ulcer index, gastric mucosal damage and histomorphology, it was found that high-, medium- and low-dose of GRR-CDs had a good inhibitory effect on acute alcoholic gastric ulcer in mice. However, there was no significant difference in gastric ulcer index and inhibition rate among the three dose groups. Therefore, we speculated that the anti-gastric ulcer activity of GRR-CDs was not dose-dependent.

As a related substance of oxidative stress, SOD is an important defense against oxidative damage [29]. MDA is the product of lipid peroxidation in the body, which can reflect the degree of lipid peroxidation [30]. Therefore, SOD and MDA were selected as indicators to evaluate the effect of GRR-CDs on oxidative stress in mice with acute alcoholic gastric ulcer. In the model group, the content of peroxides in serum and gastric tissue increased, and the activity of antioxidant enzymes and antioxidant capacity decreased, while the oxidative stress ability of GRR-CDs high-, medium- and low-dose groups mice was improved. No is a neurotransmitter and messenger molecule released by non-cholinergic and non-adrenergic nerves in gastrointestinal tract, and alcoholic gastric ulcer is usually related to the abnormal regulation of NO pathway [31,32]. High-, medium- and low-dose of GRR-CDs could significantly improve the NO level in gastric tissue of mice and kept it in normal. However, GRR-CDs had no effect on the NO content in serum, which was difficult to explain. The results showed that GRR-CDs could reduce the damage of gastric mucosa caused by free radicals in the process of alcohol metabolism by improving the antioxidant capacity of the body.

This study is a preliminary evaluation of the antiulcer effect and mechanism of the GRR-CDs, and further investigations are needed to elucidate the deeper underlying mechanisms of these effects. Moreover, the discovery of CDs in GRR and the demonstration of its antiulcer activity in this article provide a new theoretical basis for scientific research and clinical practice.

## 4. Materials and Methods

### 4.1. Materials

The GRR was purchased from Beijing Qiancao Herbal Pieces Co., Ltd. (Beijing, China). Dialysis membrane with a 1000 Da molecular weight was purchased from Beijing Ruida Henghui Science and Technology Development Co., Ltd. (Beijing, China).

The cell counting kit (CCK)-8 was purchased from Dojindo Molecular Technologies, Inc (Kumamoto, Japan). Immunoplates (96-well) were purchased from Corning Incorporated (NY, USA). The malondialdehyde (MDA), superoxide dismutase (SOD) and nitric oxide (NO) ELISA kits were supplied by Shanghai Lianshuo Biological Technology Co, Ltd. (Shanghai, China). All other commercial chemicals used in this study were analytical reagent grade and obtained from Sinopharm Chemical Reagents Beijing Co., Ltd. (Beijing, China). Deionized water was used in all experiments.

### 4.2. Animals

All the studies on animals were approved by the animal experiment ethics committee of Beijing university of Chinese medicine in accordance with the Guidelines for the Care and Use of Laboratory Animals (The ethics code is BUCM-4-2018101601-4006). Male Kunming mouse with SPF grade, weighing 30.0 ± 2.0 g, were purchased from Beijing Vital River Laboratory Animal Technology Co., Ltd. (Beijing, China). All animals were adaptive bred for one week before the experiment with a temperature 24 ± 1 °C and a relative humidity 56–65% in the well-ventilated and suitable illumination.

### 4.3. Preparation of GRR-CDs

1000 g crude GRR was placed in a crucible and calcined using the muffle furnace (TL0612, Beijing Zhong Ke Aobo Technology Co., Ltd., Beijing, China). The temperature programmed of the muffle furnace was set as follows: In the first stage, the calcined temperature was increased to 75 °C within 5 min and maintained for 25 min; In the second stage, the temperature was increased to 375 °C within 25 min and maintained for 1 h. The calcined GRR was crushed into powder after cooled to room temperature.

GRR-CDs were prepared by using dialysis method. First, adding the processed GRR powder to thirtyfold deionized water and boiled them three times for 90 min each time, while the solution was collected and concentrated after filtered with 0.22 μL organic microporous membrane. Next, the concentrated decoction was dialyzed against deionized water using a 1000 Da molecular weight cut-off (MWCO) dialysis membrane, while the deionized water out of membrane should be replaced in time during the dialysis until colorless and transparent. Finally, the solution in the membrane was collected and centrifuged at 5000 r/min for 30 min, and the supernatant was stored in refrigerator.

### 4.4. Characterization of GRR-CDs

The morphology, size, and microstructure of the synthetic GRR-CDs were characterized by transmission electron microscopy (TEM) (Tecnai G2 20, FEI Co., Hillsboro, OR, USA) at an accelerating voltage of 100 kV, while the structural details and the atomic lattice fringes of GRR-CDs were examined by high-resolution transmission electron microscopy (HRTEM) (JEN-1230, Japan Electron Optics Laboratory, Tokyo, Japan).

X-ray diffraction (XRD) (D8-Advanced X-ray diffractometer, Bruker AXS, Karlsruhe, Germany) was performed with Cu K alpha radiation (wavelength λ = 1.5418 Å).

The excitation and emission spectrum of GRR-CDs were determined by a fluorescence spectroscopy (FL) (F-4500, Tokyo, Japan). The absorption spectra of GRR-CDs was detected by a ultraviolet-visible spectrophotometer (UV-vis) (CECIL, Cambridge, UK). In addition, the Fourier transform infrared spectrum (FT-IR) (Thermo Fisher, Fremont, CA, USA) was recorded to identify the organic functional groups in GRR-CDs within a spectral window of 400–4000 cm^−1^.

The element composition, content and surface active group of GRR-CDs were analyzed by X-ray photoelectron spectroscopy (XPS) (ESCALAB 250Xi, Thermo Fisher Scientific, Fremont, CA, USA).

### 4.5. Fluorescence Quantum Yield of GRR-CDs

The fluorescence quantum yield (FQY) of GRR-CDs were determined with 0.1 M quinine sulfate as standard solution (quantum yield was 54%). In order to reduce the effect of reabsorption, the absorbance of quinine sulfate and GRR-CDs at 366 nm was kept below 0.05. The FQY of the sample was obtained by comparing the integral area of fluorescence intensity between the two. The calculation formula is as follows:

(1) $$Q_{C}=Q_{R}\times \frac{I_{C}}{I_{R}}\times \frac{A_{R}}{A_{C}}\times \frac{\eta^{2}{}_{C}}{\eta^{2}{}_{R}}$$

where “*Q*” is the abbreviation of fluorescence quantum yield, “*I*” is the integral area of emission intensity, “*A*” is the absorbance value at excitation wavelength, and “*η*” is the refractive index of solvent. The subscripts *C* and *R* represent GRR-CDs and standard, respectively.

### 4.6. Fingerprint Analysis of GRR and GRR-CDs by High Performance Liquid Chromatography

Aqueous solutions of GRR and GRR-CDs were initially prepared and processed using the same detection conditions. The fingerprint analysis in this study was performed on an Agilent series 1260 high performance liquid chromatography (HPLC) instrument (Agilent Technologies, Waldbronn, Germany) which equipping with a quaternary pump, a diode-array detector, an auto sample and a column compartment. Chromatographic separation was achieved on a Reliasil-C18 column (250 mm × 4.6 mm; 5 μm, Orochem, IL, USA) with a mobile phase consisted of acetonitrile (A) and 0.026% phosphoric acid (B), the gradient elution procedure was as follows: 5–25% A at 0–20 min; 25–40% A at 20–30 min; 40–60% A at 30–50 min; 60% A at 50–65 min. The column temperature was 25 °C, the flow rate of mobile phase was 1 mL/min, the detection wavelength was 230 nm and the injection volume was 10 μL.

### 4.7. Cytotoxicity Test of GRR-CDs

CCK-8 assay was used to investigate the effect of GRR-CDs on cytoactivity. The RAW264.7 cells were cultured in Dulbecco’s modified Eagle’s medium (DMEM) containing 20% foetal bovine serum and 1% penicillin–streptomycin double resistance in a humidified 5% CO_2_ atmosphere at 37 °C. The experiment began when the cells proliferated to 80% confluence.

First, the cells were inoculated into 96-well plates at a density of 1 × 10^4^ cells per well with 100 μL medium and incubated for 24 h in an incubator. Next, the original medium in each well was discarded after the cells adherent growth, and the cells were incubated for 24 h in the medium with GRR-CDs concentrations of 5000, 2500, 1250, 625, 312.5, 156.25, 78.1, 39 and 19.5 μg/mL, respectively. The controls were treated only with medium. Then, the culture medium containing the drug was washed with PBS, and the detection solution in CCK-8 detection kit was added into each well. The 96-well plates were incubated for 3 h at 37 °C. Finally, the optical density (OD) of each well was measured at 450 nm wavelength by a microplate reader (BioTek, VT, USA), and the relative cell activity was calculated. The calculation formula was as follows:

(2) $$\mathrm{Cell}\;\mathrm{viability}\;\left(\%\;\mathrm{of}\;\mathrm{control}\right)=\frac{{\mathrm{Abs}}_{\mathrm{sample}}}{{\mathrm{Abs}}_{\mathrm{control}}}\times 100$$

Abssample and Abscontrol represent the A450 of the experimental groups and control group, respectively. The experiments were performed in triplicate, independently.

### 4.8. Study on Anti-Ulcer Activity of GRR-CDs

The dose-effect relationship of GRR-CDs were investigated in the mice model with acute alcoholic gastric ulcer. Male Kunming mice with SPF grade were randomly divided into five groups (*n* = 9 per group): control group (CG), model group (MG), GRR-CDs high-dose group (CHG), GRR-CDs medium-dose group (CMG) and GRR-CDs low-dose group (CLG). After 8 h of fasting, all groups mice except the control were given a dose of 10 mL/kg of 70% alcohol by intragastric gavage to establish the acute alcoholic gastric ulcer model, while the control group was given equal volumes of normal saline. After an hour, the high-, medium- and low-dose groups were given GRR-CDs with 9 mg/kg, 6 mg/kg and 3 mg/kg by intragastric gavage, respectively. While the control and model group were given equal volumes of normal saline. The above steps were repeated for five days.

Twenty four h after the last administration, the whole blood of mice was collected from the orbit. After death by cervical dislocation, the abdominal cavity of the mice was opened, and the gastric tissue was removed and cut open. The inner surface of the gastric was scanned to observe the damage and calculate the gastric ulcer index. Half of the gastric tissue was fixed, embedded, sectioned and stained for histopathological examination, while the serum and the other half of the gastric tissue were used for further study.

Gastric ulcer index: When the gastric bleeding was speckled (less than 1 mm in length and width), 1 point was counted; when threadiness, 2 points for less than 1 mm and 3 points for 1–2 mm; when plaque (length and width are both greater than 2 mm), the area was calculated.

Gastric ulcer inhibition rate = [(gastric ulcer index of model group − gastric ulcer index of experimental group)/gastric ulcer index of model group] × 100%

### 4.9. Detection of Biochemical Indexes in Serum and Tissues

The whole blood was put into the EP tube and left for 30 min, then the upper serum was collected after centrifuged at 3000 r/min for 10 min.

The gastric tissue was weighed and placed into the EP tube with 9 times volume of normal saline. The gastric tissue homogenate was prepared by an internal tissue homogenizer (FLUKO Equipment Shanghai Co., Ltd., China) and centrifuged at 3000 r/min for 15 min. The supernatant was collected.

The content of malondialdehyde (MDA), superoxide dismutase (SOD) and nitric oxide (NO) in the serum and the gastric tissue were detected by using ELISA kits according to the instructions, respectively.

### 4.10. Statistical Analysis

The experiment results were expressed as the mean ± standard deviation (SD), and the comparisons among groups were performed by using the single factor analysis of variance (ANOVA) and the least significant difference (LSD) in Statistic Package for Social Science software (SPSS 22.0, Inc., Chicago, IL, USA). The *p* value < 0.05 was considered statistically significant for the analyses.

## 5. Conclusions

This study is the first to report that GRR-CDs, the new substance produced during the high temperature carbonization of GRR, are the main active compounds in the anti-gastric ulcer effect of GRR. It is found that GRR-CDs are the spherical structure with a large number of active groups on their surface and the particle size ranged from 1 to 5 nm by electron microscopy and spectroscopy characterization. The absence of cytotoxicity of GRR-CDs indicates that they have a good biological application prospect. The present study is a preliminary evaluation of the antiulcer effect and mechanism of the GRR-CDs, which might contribute to the development of potential treatment strategies for acute alcoholic gastric ulcer. The results provide a new perspective and innovative method for the material basis study of GRR. In addition, it also provides a new theoretical basis for scientific research and clinical practice.

## Figures and Tables

**Table 1:** Comparison of anti-gastric ulcer activity of different doses of GRR-CDs.

Groups	Gastric Ulcer Index	Gastric Ulcer Inhibition Rate (%)
control	0	100
model	47.00 ± 4.60 ^#^	0
GRR-CDs high-dose	18.17 ± 3.81 *	61.34
GRR-CDs medium-dose	12.83 ± 3.97 *	71.17
GRR-CDs low-dose	15.17 ± 3.37 *	67.72

# *p* < 0.01 compared with control group; * *p* < 0.01 compared with model group.

## Data Availability

The data presented in this study are available on request from the corresponding author.

## Sample Availability

Samples of the compound GRR-CDs is available from the authors.
